# Metal–Organic Framework/Polyvinyl Alcohol Composite Films for Multiple Applications Prepared by Different Methods

**DOI:** 10.3390/membranes13090755

**Published:** 2023-08-24

**Authors:** Binyan Liu, Shuhua Zhang, Ming Li, Yu Wang, Dajiang Mei

**Affiliations:** College of Chemistry and Chemical Engineering, Shanghai University of Engineering Science, Shanghai 201620, China; m040120232@sues.edu.cn (B.L.); m040120321@sues.edu.cn (M.L.); m340121234@sues.edu.cn (Y.W.)

**Keywords:** metal–organic frameworks, polyvinyl alcohol, film

## Abstract

The incorporation of different functional fillers has been widely used to improve the properties of polymeric materials. The polyhydroxy structure of PVA with excellent film-forming ability can be easily combined with organic/inorganic multifunctional compounds, and such an interesting combining phenomenon can create a variety of functional materials in the field of materials science. The composite membrane material obtained by combining MOF material with high porosity, specific surface area, and adjustable structure with PVA, a non-toxic and low-cost polymer material with good solubility and biodegradability, can combine the processability of PVA with the excellent performance of porous filler MOFs, solving the problem that the poor machinability of MOFs and the difficulty of recycling limit the practical application of powdered MOFs and improving the physicochemical properties of PVA, maximizing the advantages of the material to develop a wider range of applications. Firstly, we systematically summarize the preparation of MOF/PVA composite membrane materials using solution casting, electrostatic spinning, and other different methods for such excellent properties, in addition to discussing in detail the various applications of MOF/PVA composite membranes in water treatment, sensing, air purification, separation, antibacterials, and so on. Finally, we conclude with a discussion of the difficulties that need to be overcome during the film formation process to affect the performance of the composite film and offer encouraging solutions.

## 1. Introduction

Polymer is a traditional material with macromolecular structure that is widely used in our daily life due to its light weight, low cost, and chemical stability [1]. The processing of polymer materials into polymer film materials that are indispensable in our lives has a wide range of applications, such as greenhouses [2,3], packaging [4], antimicrobials [5], and agricultural films [6,7]. To further expand the applications of polymeric film materials, and to obtain greater performance advantages than can be obtained by using each component alone, solid fillers and additives are often added to produce mixed matrix membranes (MMMs) [8]. MMMs typically consist of a solid filler (dispersed phase) and a processable polymer matrix (continuous phase). Typical MMMs are made by incorporating various zeolites [9], activated carbon [10], molecular sieves [11], metal oxides [12], non-porous silica [13], and other traditional inorganic fillers into the polymer matrix. However, these conventional fillers often result in non-selective voids or defects in MMMs due to poor polymer–filler compatibility. 

With the rapid development of materials science and technology, research in recent years has shifted to porous materials combining rich chemical functionality and tunable pore structure, such as metal–organic framework materials (MOFs), covalent organic frameworks (COFs), and porous organic cages (POCs), to prepare defect-free and well-performing MMMs, which has also greatly broadened the range of potential fillers in MMMs [14]. Among them, MOFs are an emerging porous material compared to traditional inorganic fillers, which are bonded by metal ions or clusters and organic linkers and are known for their huge specific surface area and ultra-high porosity coupled with the variability of the organic and inorganic components in their structure, where the sum of the physical properties of the inorganic and the organic components and the possible synergistic interactions between the two provide MOFs with interesting properties [15,16]. Currently, most of the synthesized or reported MOFs are in the form of powder, and MOF powder is prone to dust generation due to its poor processing properties, and some processing problems associated with MOF powder have hindered its industrial application in mass production, so there is a need to develop reliable methods to apply MOF powder in practical applications, such as in the photocatalytic reduction of CO_2_, where MOF powder has the drawbacks of light scattering and being difficult to recycle after the reaction, which has limited its practical application [17]. The simplest method usually requires mixing and granulation of MOF powders with binders, but such methods reduce the internal surface area and cause pore blockage [18]. Another approach is to combine MOFs with other materials to form composites, which gives more functions to MOF-based composites through synergistic effects [19].

Reports on MOF-based thin film materials exhibit many excellent physical and chemical properties [20,21]. If an inorganic substrate is used in the preparation of the composite film, the obtained film has sufficient mechanical strength, chemical stability, and high-temperature resistance. However, the inorganic substrate preparation process is cumbersome, expensive, brittle, and difficult to implement on a large scale, which limits its wide application. Comparatively, polymer membranes have the advantages of low cost, large membrane area, high processing capability, and large-scale industrial applications [22]. Many polymer materials, including polyacrylonitrile (PAN) [23], polylactic acid (PLA) [24,25], polyvinylpyrrolidone (PVP) [26], polyamide (PA) [27], and other polymers, are used as substrates for the preparation of MOF-based composite membranes, which have a wide range of applications in the fields of water treatment, antimicrobials, air purification, sensing, etc. For example, Sorribas et al. [28] synthesized composite membranes containing ZIF-8, MIL-53(Al), NH_2_-MIL-53(Al), and MIL-101(Cr) in a PA film layer by in situ interfacial polymerization with high flux nano-filtration of organic solvents. Zhang et al. [29] embedded Ag_2_(HBTC) into PLA fiber membranes as an antibiotic-free material by an electrostatic spinning process, which was effective in killing bacteria. Among them, polyvinyl alcohol (PVA) has excellent oil and chemical resistance and also good biodegradability, making it an attractive bioplastic material [30,31]. PVA, with its excellent film-forming ability and its films, is considered to be a smart material that can be functionally tailored according to the usage interest due to its colorless and transparent, non-toxic and non-hazardous, good physicochemical properties and biodegradability [32]; thus, it has a wide range of applications in the fields of catalysis [33], controlled release of drugs [34], noise reduction [35,36], food packaging [37], and so on. These PVA films will be well used in one aspect due to their high hydrophilicity; however, in most cases, the high hydrophilicity will hinder their application in aqueous environments. To improve the stability and properties of PVA films, various physicochemical methods have been used, such as cross-linking [38], electrostatic spinning [39], blending [40], sol–gel processing [41], and grafting [42].

The polyhydroxy structure of PVA can be easily combined with organic/inorganic multifunctional compounds to create a variety of functional materials in the field of materials science. The composite film obtained by combining MOFs with PVA is a composite material consisting of dispersed phases (MOFs) and continuous phases (PVA), the organic ligands in the MOFs of this composite have good affinity and compatibility with the polymer PVA chains, and the strong interactions with each other can combine the processability of PVA with the excellent properties of porous filler MOFs to maximize the advantages of the material [43]. In addition, the combination of multifunctional MOFs and PVA with a polyhydroxy structure can not only solve the problems of instability and difficulty in recycling single-use MOFs but also improve the physicochemical properties of PVA and expand a wider range of applications. However, in the process of preparing MOF/PVA composite film, the MOF particles may be covered by PVA or agglomerate due to the high surface energy of MOFs, resulting in a decrease in active sites and specific surface area. Therefore, we will summarize and analyze in a more systematic way the methods used to prepare MOF/PVA composite films and their applications in different directions, as shown in Table 1, and also propose encouraging solutions to the challenges faced to further their functionalization.

## 2. Preparation of MOF/PVA Composite Film

MOF/PVA composite film preparation methods can be widely divided into two categories: solution casting and electrostatic spinning; in addition, spraying, spin coating, and vacuum filtration methods can be used to prepare MOF/PVA composite film. Depending on the preparation process of MOF combined with PVA, it can be further divided into MOF and PVA blending, in situ growth, and layer-by-layer assembly of MOF particles on PVA. The comparison of various MOF/PVA composite film preparation methods is shown in Table 2.

### 2.1. Solution Casting

**Figure 1:**
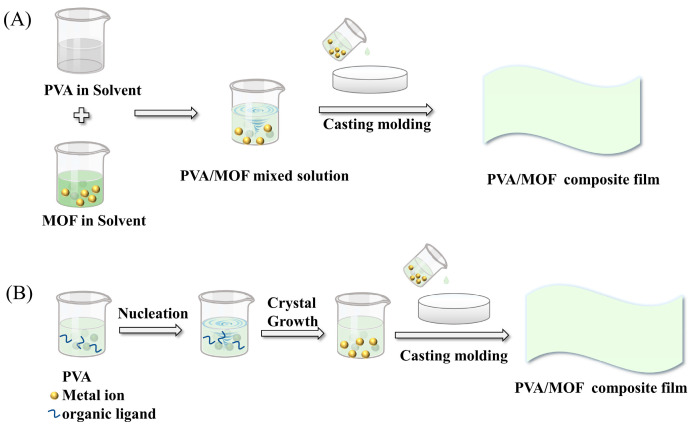
Schematic diagram of the process of preparing MOF/PVA composite film materials by solution casting. (**A**) Pre-synthesised MOF particles blended with PVA. (**B**) In situ growth of MOF particles in PVA.

Solution casting is a simple solution blending method; the usual method is to dissolve the polymer in a solvent, and then the inorganic nanoparticles are dispersed in the solvent through certain physical methods to make the nanoparticles uniformly dispersed in the polymer solution, and finally the mixed solution containing nanoparticles is poured into the mold and dried to obtain a composite material. The solution casting method is easy to operate and low cost. For the preparation of PVA and MOF particles by the solution casting method to obtain MOF/PVA composite film material, the process is schematically shown in Figure 1, which is the blending of MOF particles with PVA and the in situ growth of MOF particles in PVA, respectively.

#### 2.1.1. Blending

**Figure 2:**
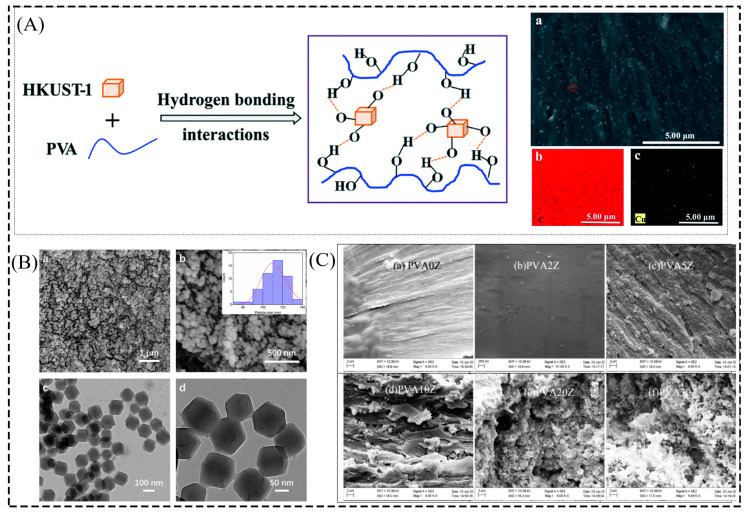
(**A**) HKUST-1 interaction process with PVA and (**a**) SEM and (**b**,**c**) EDX images. (**B**) (**a**,**b**) SEM and (**c**,**d**) TEM images of ZIF-8 nanoparticles. (**C**) FE-SEM images of fractured surfaces of ZIF-8/PVA composite films. (**a**–**f**) Denote different levels of ZIF-8 in the composite film. Reprinted with permission from Refs. [45,47,48]. Copyright 2018 The Royal Society of Chemistry. Copyright 2021 Walter de Gruyter GmbH. Copyright 2016 The Royal Society of Chemistry.

The most important challenges in preparing composite films are the strong interfacial interactions between the organic and inorganic phases and the homogeneous dispersion of the inorganic particles [44]. Similarly, the key to preparing MOF/PVA composite films by a simple and low-cost solution casting method is the effective dispersion of MOF particles in the casting solution to achieve a defect-free MOF-PVA interface. The PVA/HKUST-1 composite film material was obtained by ultrasonically dispersing the HKUST-1 particles in ethanol and mixing them with the PVA solution. SEM and EDX plots showed that HKUST-1 was uniformly dispersed in the composite film and that the composite film exhibits good thermal, mechanical, and UV-blocking properties (Figure 2A) [45]. Another approach is to modify the MOF particles with functional groups to tune their surface properties and thus improve their adhesion to the polymer [73,74]. Jiang et al. [46] prepared UiO-66-X particles with different ligand modifications and then mixed them with a PVA solution to obtain UiO-66-X/PVA hybrid films. The SEM images showed that the UiO-66-X particles modified with different ligands were uniformly loaded on the UiO-66-X/PVA hybrid membrane. The cross-sections of all the membranes showed dense morphology, which could be further applied in osmotic dehydration separation. Therefore, the MOF particles are first dispersed in the solvent by a certain method. Then, the suspension is slowly reacted with the PVA solution to facilitate the homogeneous dispersion of the MOF particles in the PVA solution. The problem of poor compatibility and particle agglomeration and deposition between the MOF filler and the PVA matrix can also be solved by adjusting the surface functional groups of the MOF particles.

The size and morphology of MOFs are also key factors affecting the performance of composite films [75]. Nanoparticles are often preferred because they can bind tightly to polymers to form a larger interfacial area [76]. ZIF-8 nanoparticles with uniform size distribution and regular polyhedral shape without obvious agglomeration were dispersed with PVA in DMF solution ultrasonically and through heating and then cast to obtain a PVA/ZIF-8 composite membrane with good mechanical properties and ionic conductivity (Figure 2B) [47]. However, ZIF-8/PVA composite films were prepared by loading non-spherical and agglomerated ZIF-8 particles onto PVA [48]. FE-SEM plots of the fractured surfaces of the composite films showed that ZIF-8 particles were uniformly distributed in the PVA matrix at 2 wt% loading in the ZIF-8/PVA composite film, but agglomeration occurs from 5 wt% loading, and the agglomeration effect was more pronounced at higher loading levels (Figure 2C). Adjusting the size and morphology of the MOF particles can also contribute to improving the performance of the composite film.

**Figure 3:**
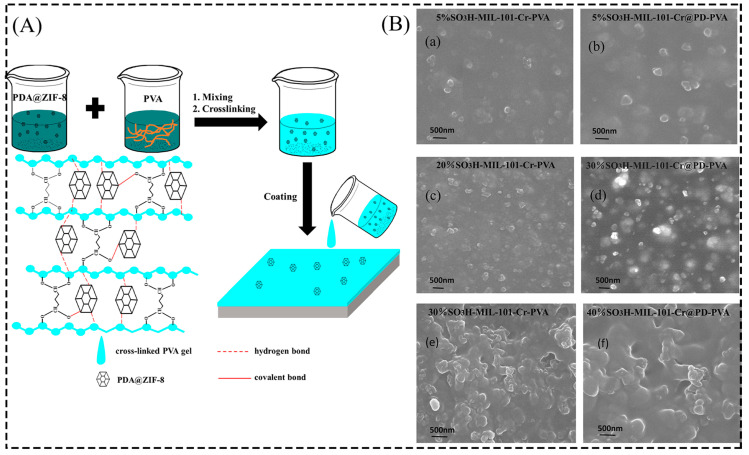
(**A**) Preparation of PVA/PDA@ZIF-8 composite film. (**B**) SEM images of the surface of SO_3_H-MIL-101-Cr-PVA film and SO_3_H-MIL-101-Cr@PD-PVA film with different loadings. (**a**,**c**,**e**) and (**b**,**d**,**f**) denote the addition of different levels of SO_3_H-MIL-101-Cr and SO_3_H-MIL-101-Cr@PD to the PVA film, respectively. Reprinted with permission from Refs. [50,51]. Copyright 2020 Elsevier Inc. Copyright 2017 Elsevier B.V.

In addition, researchers have attempted to improve the performance of composite films by adding dispersants and cross-linkers to the reaction solution to improve the good affinity between the filler and polymeric materials, enabling the formation of ideal defect-free composite films [46,49]. Fu et al. [50] added the dispersant polydopamine (PDA) to the solution and obtained homogeneous PVA/PDA@ZIF-8 composite membranes with strong antifouling properties and permeation (Figure 3A). Zhang et al. [51] also used the dispersant PD during the reaction and obtained homogeneous discrete SO_3_H-MIL-101-Cr@PD nanoparticles. In addition, they studied hybrid films formed with different filler amounts of PVA in the presence of cross-linkers with polyacrylonitrile (PAN) film carriers and found that the SO_3_H-MIL-101-Cr@PD particles were uniformly distributed in each hybrid film even at a loading of 30 wt% (Figure 3B). In contrast, hybrid films loaded with 30 wt% SO_3_H-MIL-101-Cr without the presence of PD showed significant agglomeration. Zhu et al. [49] observed a significant agglomeration of ZIF-90 particles in the ZIF-90/PVA-10 composite film without the presence of the cross-linker APTES by SEM maps. In contrast, the ZIF-90 particles cross-linked with APTES exhibited better dispersion in the composite membrane and showed high dehydration performance for ethanol separation. In addition, the uniformity of the particles was greatly improved when the composite films obtained by cross-linking different levels of Cu MOF with PVA in the presence of glutaraldehyde (GA) were compared with those without cross-linking agents [52]. By comparing with the presence of a cross-linking agent and a dispersant in the solution, it can be seen that the presence of a cross-linking agent and a dispersant promotes the effective dispersion of MOF particles in the composite film and helps to improve the performance of the composite film.

#### 2.1.2. In Situ Growth

**Figure 4:**
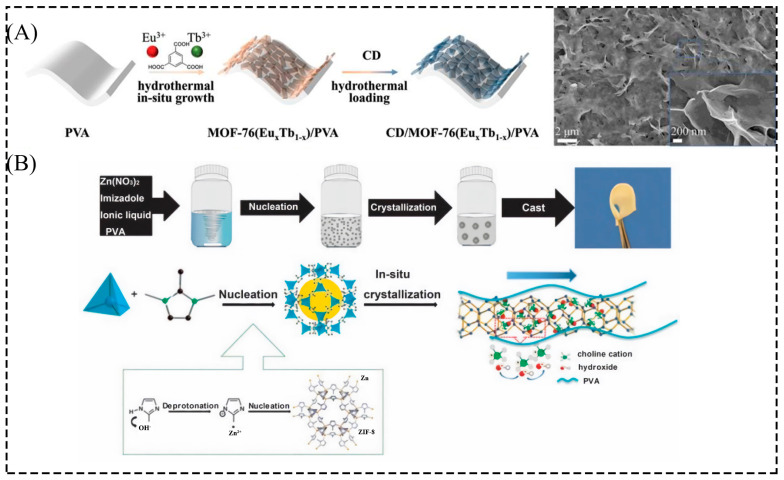
Schematic diagram of (**A**) the preparation of CD/MOF-76(Eu_x_Tb_1−x_)/PVA composite film and (**B**) the preparation of ZIF-8/PVA composite film. Reprinted with permission from Refs. [53,55]. Copyright 2021 The Royal Society of Chemistry. Copyright 2014 The Royal Society of Chemistry.

By in situ growing MOF particles in a polymer solution to prepare a composite film, MOF particles can be evenly and size-controllably dispersed in the polymer matrix, which can well solve the problem of aggregation of MOF fillers in the polymer solution. Water-soluble polymers have been reported to positively affect the dispersion of nanoparticles during in situ synthesis [77]. Li et al. [53] grew MOF-76(Eu_x_Tb_1−x_) and loaded CD in situ on the PVA membranes by the hydrothermal method. SEM plots showed that CD and MOF-76(Eu_0.05_Tb_0.95_) grew in abundant and uniform porous sheets on the PVA surface, which in turn increased the binding force between MOF and PVA, allowing the composite films to exhibit high thermal and mechanical stability, as well as showing exceptional sensitivity in detecting CBZ at the nanoscale (Figure 4A). In addition, the reaction of a solution containing PVA, metal cations, and organic ligands has resulted in a ZIF-90/PVA composite membrane that can be applied to the osmotic dehydration of ethanol. Moreover, as the viscosity of the PVA solution increases, the size of the ZIF-90 particles decreases, which further prevents the aggregation of ZIF-90 particles in the system [54]. Therefore, the viscosity of the PVA solution also contributes to the regulation of the size and dispersibility of the MOF particles. In addition, Liu et al. [55] added PVA into a fully dissolved solution containing metal cations and organic ligands, allowing the ZIF-8 particles to grow in situ in the PVA (Figure 4B). TEM and PXRD showed that in situ crystallization was very effective for preparing ZIF-8/PVA homogeneous composite films. Therefore, a composite film of MOF particles grown in situ in PVA is obtained, either by placing the PVA film in a solution containing metal cations and organic ligands or by combining the PVA of the solution with a solution containing metal cations and organic ligands in situ, and the viscosity of the PVA solution also influences the in situ crystallization process of the MOF particles. 

### 2.2. Electrostatic Spinning

**Figure 5:**
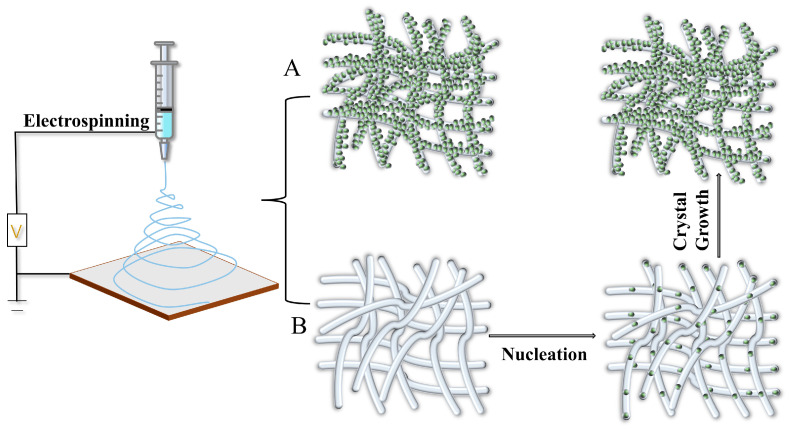
Schematic diagram of the process of preparing MOF/PVA composite film by electrostatic spinning. (**A**) Pre-synthesised MOF particles blended with PVA. (**B**) Layer-by-layer assembly of MOF particles on PVA fibre film.

Electrostatic spinning involves the application of a high potential (typically 10–30 kV) to a polymer solution or polymer melt with a certain viscosity. The high potential causes the polymer to be rapidly stretched and twisted after the solvent evaporates, eventually forming microns/nanofibers attached to a specified substrate [78,79]. Nanofibers have fascinating properties such as a high aspect ratio, large specific surface area, multi-sized porosity, and high flexibility. Compared to conventional methods of preparing thin film materials, electrostatic spinning is a low-cost, simple, high-throughput, high-porosity, and high-mechanical-strength method of film preparation. It has been reported that various MOF materials can be molded into hybrids with multi-sized porosity and multifunctionality by combining them with electrostatically spun polymers [80]. The organic part of the MOF and the electrostatically spun polymer are usually compatible, which makes it possible to distribute the MOF material evenly at high loading rates, making it possible for the mixture to aggregate less [81]. For the preparation of PVA and MOF particles by electrostatic spinning to obtain MOF/PVA composite film materials, the process is schematically shown in Figure 5, where the pre-synthesized MOF particles are blended with PVA, and the MOF particles are assembled layer by layer on the PVA film, respectively.

#### 2.2.1. Blending

**Figure 6:**
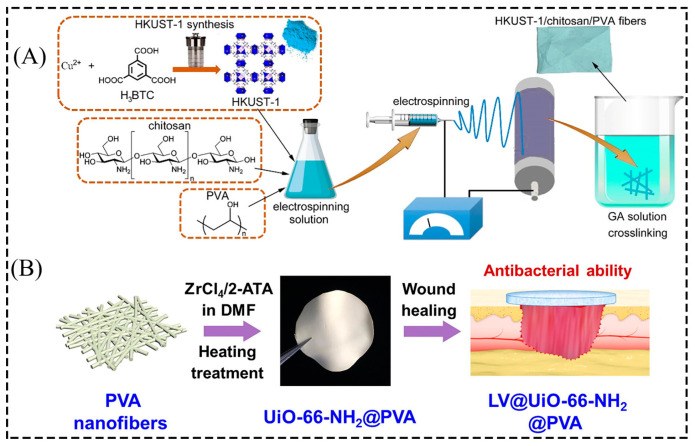
(**A**) Schematic diagram of the HKUST-1/chitosan/PVA fiber membrane preparation process. (**B**) Schematic diagram of the preparation process of UiO-66-NH_2_@PVA nanofiber membrane. Reprinted with permission from Refs. [57,58]. Copyright 2020 Elsevier B.V. Copyright 2021 Elsevier Inc.

The MOF/PVA nanofiber membrane is usually made by mixing pre-synthesized MOF particles with a PVA solution and then electrostatic spinning. Lou et al. [56] prepared MOF-5-NH_2_/PVA nanofiber membranes with different loadings by electrostatic spinning by blending MOF-5-NH_2_ nanoparticles with a PVA solution. This nanofiber membrane had good structural stability, flexibility, and potential for gas sensing and detection applications. In addition, the researchers have improved the performance of the composite fiber membrane by adding a cross-linking agent to the reaction system to improve the effective binding of the MOF particles to the PVA. Wang et al. [57] reported the preparation of HKUST-1/chitosan/PVA fibrous membranes with antibacterial properties by electrostatic spinning and a cross-linking process (Figure 6A). The SEM diagram showed that the chitosan/PVA fiber membrane had a smooth surface, and the EDS diagram shows a uniform distribution of Cu elements in the fiber, indicating that the MOF particles were uniformly incorporated in the fiber. Therefore, MOF particles can be uniformly and effectively combined with PVA by the cross-linking method.

#### 2.2.2. Layer-by-Layer Assembly

By in situ layer-by-layer growth of MOF particles in nanofibers, higher specific surface area and active sites can be obtained, which helps improve the composite film’s performance. Zhu et al. [58] reported that PVA nanofiber membranes were first prepared by electrostatic spinning. The PVA nanofiber membranes were placed in a solution containing metal cations and ligands. Finally, MOF particles were densely nucleated on the surface of the PVA nanofiber membranes to obtain UiO-66-NH_2_@PVA nanofiber membranes with antibacterial properties (Figure 6B). In addition, to overcome the problem that the PVA fiber film obtained by electrostatic spinning is unstable because it is water-soluble, Truong et al. [59] investigated ways such as cross-linking to make it more stable. Before electrostatic spinning, citric acid, maleic acid, and polyacrylic acid (PAA) were added to the PVA solution to prepare cross-linked PVA electrostatic spinning films. Then, the stable PVA films were sequentially immersed in a solution containing metal cations and organic ligands, cycling the immersion five times to allow in situ growth of HKUST-1 on the surface of the stable PVA films. The presence of HKUST-1 particles on all three PVA cross-linked electrostatic spinning membranes was observed by SEM images, with better growth of HKUST-1 particles on the cross-linked citric acid–PVA membrane and the potential of this fiber membrane for air purification applications.

### 2.3. Alternative Methods

**Figure 7:**
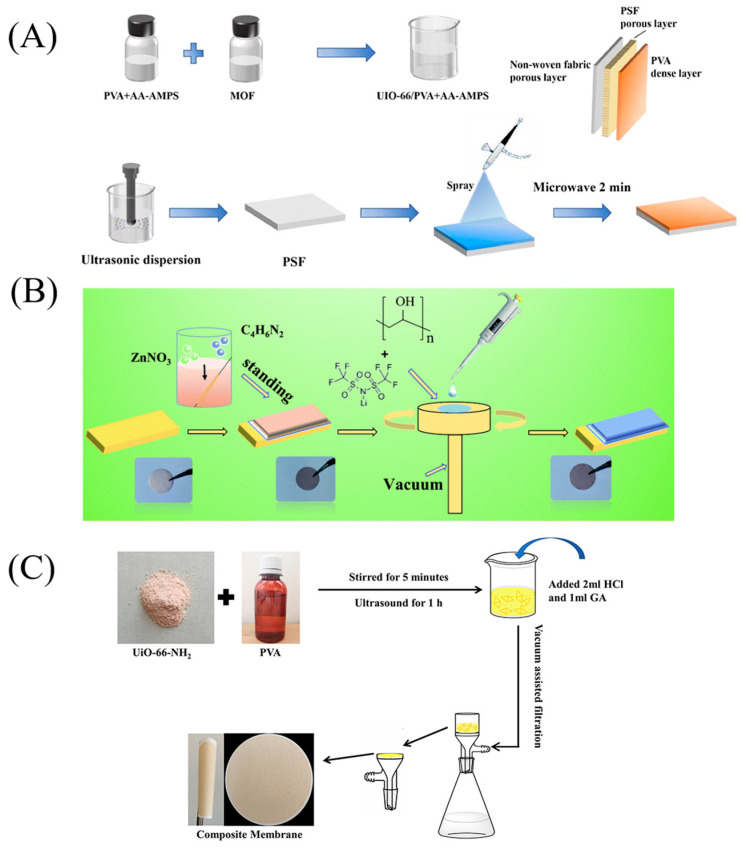
Schematic diagram of the preparation of (**A**) PVA-P(AA-AMPS)-UIO-66 composite film. (**B**) Man-made SEI film. (**C**) UiO-66-NH_2_/PVA bilayer composite membrane. Reprinted with permission from Refs. [60,61,62]. Copyright 2022 Elsevier B.V. Copyright 2020 The Royal Society of Chemistry. Copyright 2022 Elsevier Ltd.

In addition to solution casting and electrostatic spinning, spraying, spin coating, and vacuum filtration methods can be used to prepare MOF/PVA composite films. To reduce the agglomeration of MOF particles, Xing et al. [60] applied a mixture of PVA, polyacrylic acid co-2-acrylamide-2-methyl propane sulfonic acid (P(AA-AMPS)), and UiO-66 by spraying onto a polysulfone substrate using an airbrush. Finally, the composite film obtained after cross-linking in a microwave oven had a smooth surface without defects, uniform distribution of UiO-66 particles, and a load rate of 30% (Figure 7A). The composite membrane also exhibited very high water flux and salt rejection even when treating concentrated brine. Fan et al. [61] first grew Zn-MOF particles uniformly in situ on the surface of copper foil at room temperature. Then, spin-coated PVA gel solutions of different viscosities were permeated onto the surface of Zn-MOF particles to obtain a Zn-MOF/PVA artificial solid electrolyte interface (SEI) film. This SEI film had high lithium-ion conductivity and good flexibility (Figure 7B). Chen et al. [62] synthesized a new bilayer composite membrane by a simple vacuum filtration method using the cross-linking agent GA to form a dense UiO-66-NH_2_/PVA layer on a PTFE membrane substrate (Figure 7C). This composite membrane showed good resistance to contamination and wetting by oil and surfactants. Such a range of other methods can also bind MOF particles to PVA substrates uniformly and effectively at high loading. When using different methods, exploring the combination of appropriate PVA solution viscosity, the addition of cross-linkers to the system and other conditions will result in better film formation.

In conclusion, the preparation of MOF/PVA composite films by electrostatic spinning is a low-cost, easy-to-operate film preparation method with high flux, porosity, and mechanical strength. This process requires a few polymer and film post-treatment processes, making electrostatic spinning a more environmentally friendly technology. Moreover, electrostatic spinning can make the MOF particles combine with PVA in a more uniform and stable manner with high loading to increase the specific surface area, porosity, and active sites of the composite film, thus giving the MOF/PVA composite film more excellent performance and expanding its wider applications.

## 3. Applications

With the development of materials science and technology, there is a strong interest in developing new porous materials with excellent properties for the needs of everyday life and the advancement of industrial applications. According to the pore size, porous materials can be divided into large pores with diameters greater than 50 nm, mesopores with diameters between 2 and 50 nm, and microporous pores with diameters less than 2 nm. Traditional porous materials have a wide range of structures from microporous to mesoporous. The limited adjustable pore structure of traditional porous materials limits the range of applications that require large pores [19]. In contrast, MOFs with typical micro- or mesoporous materials can be easily adjusted to meet practical requirements by changing the metal source, organic ligand, reaction solvent, templating agent, and temperature to adjust the pore size and design a specific morphology of the MOF material [82]. Among these, surfactants have been selected as novel templating agents for the synthesis of mesoporous MOFs due to their outstanding performance in the preparation of mesoporous molecular sieve materials [83,84]. This is demonstrated by the tendency of the MOF precursors to nucleate spontaneously at the edges of the surfactant during the crystallization process, resulting in the formation of MOFs with adjustable pore size, micro- and mesopores, and specific morphology. Such MOF materials with their high crystallinity, tunable composition structure, high porosity, and large surface area make them promising for other fields such as gas storage [85], separations [86,87], adsorptions [88], sensing [89,90], and drug delivery [91,92].

MOFs, porous materials consisting of inorganic nodes connected to organic ligands by coordination bonds, exhibit relatively low stability compared to conventional porous materials (e.g., zeolites or porous carbon-based materials) under mostly weaker coordination bonds than covalent bonds [93]. The processing properties of highly crystalline MOFs are not yet satisfactory, and generally, the synthesized or reported MOFs are obtained in the form of tiny crystals of tens to hundreds of microns or powder form for large samples. However, in practical applications, the microscopic morphology, structure, mechanical properties, and other physicochemical properties of the material are of great importance [94]. MOF powder hinders its further large-scale application due to poor mechanical processing properties, poor mass and heat transfer rates, and easy dust generation, e.g., in the photocatalytic reduction of CO_2_, MOF powder has disadvantages such as light scattering and difficulty in recycling after the reaction, which limits its practical application [17]. Therefore, reliable methods need to be developed to mold MOF powders into formed products with better performance for practical applications without significantly sacrificing the inherent properties of the original MOF samples. The simplest method usually involves mixing and granulating MOF powder with a binder, but such methods reduce the internal surface area and cause pore blockage [95]. Another approach is to combine MOFs with other materials to form composites, giving more functionality to MOF-based MMMs through synergistic effects [96]. Membrane preparation from a polymer matrix is the most extensive method, and a polymer membrane has a wide range of applications in fuel cells, biomedicine, and separation [97,98]. PVA membrane materials have attracted wide attention due to their easy processing, low cost, non-toxicity, and biocompatibility. The combination of PVA and MOF materials with a variety of excellent properties allows the processing of ideal materials with a variety of properties, making MOF/PVA composite film materials have a wide range of applications in water treatment, sensing, air purification, antibacterials, UV blocking, and other fields.

### 3.1. Water Treatment

Discharging wastewater containing pollutants into the natural environment is one of the major causes of water scarcity. To date, many scientists have used different water treatment technologies (membrane treatment, adsorption, advanced oxidation, etc.) to mitigate environmental pollution [99,100]. Due to its large pore surface area, micro- and mesopores, adjustable structure, and ability to interact with specific adsorbents/sorbents [101], MOFs have attracted a great deal of interest from researchers in the area of high-capacity adsorbents [102], removal of organic pollutants [103], heavy metal removal [104], and other aspects of water purification. Different types of MOF materials are used for water treatment applications, e.g., water-stable MOFs, defective MOFs, and layered pore MOFs [105,106]. Typically, defective MOFs have higher porosity and surface area, contributing to the formation of defective active sites and thus enhanced adsorption properties. Water-stable and multi-dimensional MOFs often exhibit high adsorption performance due to their high structural stability, high specific surface area, high porosity, and high channeling that can match organic pollutants.

**Figure 8:**
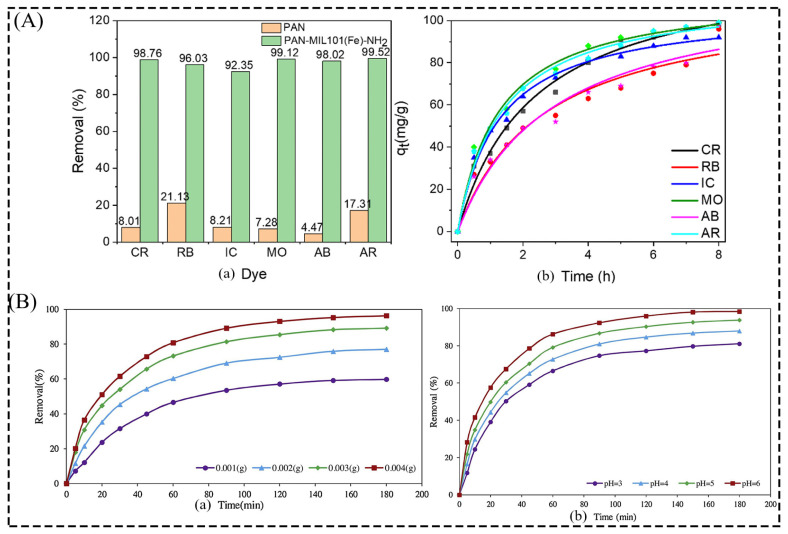
(**A**) (**a**) Dye removal efficiency and (**b**) adsorption kinetics of PAN and PAN-MIL101(Fe)-NH_2_ NFM; (**B**) (**a**) effect of different dosages of ZIF-8@CS/PVA-ENF on MG dye removal (%); (**b**) effect of solution pH on dye removal (%) at optimum dosage. Reprinted with permission from Refs. [63,108]. Copyright 2022 MDPI AG. Copyright 2019 Elsevier Ltd.

In addition, the assembly of MOFs suitable for water treatment into thin film materials helps to improve the effectiveness and efficiency of many industrial processes, such as wastewater purification, and avoids the problems of difficult recovery and secondary contamination caused by powdered MOFs [107]. MOFs have better compatibility with polymeric matrices than other inorganic porous materials, resulting in significantly improved selectivity and permeability in water treatment processes. Jia et al. [108] prepared PAN/MIL101(Fe)-NH_2_ nanofiber membranes (NFMs) by a one-step electrostatic spinning method. By comparing the adsorption performance of pure PAN membranes, powder MIL101(Fe)-NH_2_, and NFM for separating dyes and acidic dye molecules in wastewater, it was shown that the NFM had better adsorption and separation performance for acidic organic ionic dyes in aqueous solution due to the synergistic effect of PAN and MIL101(Fe)-NH_2_ crystals (Figure 8A). A mixture of poly(styrene-alt-maleic-anhydride) (PSMA) and UiO-66-NH_2_ was prepared by solution casting to obtain cross-linked MOF/PSMA composite membranes with different MOF contents by Cao et al. [109]. This MMM has high adsorption capacity for the separation of methylene blue (MB) and methyl orange (MO), common organic dye pollutants in wastewater. In addition to this, the polymeric substrates currently used to combine with MOF materials to form MMM and for water treatment are polyamide (PA) [110], polyacrylic acid (PAA) [111], polyvinylidene fluoride (PVDF) [112], polyethersulfone (PES) [113], PVA, etc. Among them, PVA [63], a biodegradable and environmentally friendly material, combined with MOF particles with a large specific surface area and tunable pore structure to obtain composites, is considered a potential active material for environmental treatment and remediation systems [114,115]. Liu et al. [116] systematically summarized the mechanism of MOF matrix membranes in the adsorption and removal of toxic and hazardous substances from water mainly through electrostatic interactions, hydrogen bonding adsorption, π–π interactions, acid–base interactions, etc., which laid the foundation for this paper to discuss the content of MOF/PVA composite membranes in water purification. The PVA-P(AA-AMPS)-UiO-66 composite membrane obtained by Xing et al. [60] prepared by a spraying method showed very high water flux and salt rejection even in the treatment of concentrated brine, which is conducive to solving the problem of water scarcity. Mahmoodi et al. [63] investigated the removal of malachite green (MG) dye by prepared ZIF-8@CS/PVA ENF through batch adsorption experiments. The increase in the effective surface area and porosity of the nanofibers increases the effective adsorption sites on the adsorbent surface and improves surface accessibility. ZIF-8@CS/PVA-ENF at an optimal dose of 0.003g and sample pH of 6 are ideal conditions to improve the maximum removal of MG from an aqueous solution (Figure 8B). In addition, ZIF-8@CS/PVA-ENF(2) after two cycles of loading ZIF-8 showed the highest reusability and stability. These water-stable and reusable MOF-based MMMs show that they are promising materials for the efficient capture of pollutants in wastewater. In recent years, a hot research topic for MOF-based MMMs in water treatment has been the preparation of high-quality, stable membranes by selecting suitable preparation methods, adjusting the structural properties of the membranes, and modifying the membrane surface to ultimately expand their application areas [117].

### 3.2. Sensing

Since chemical sensing performance is mainly influenced by surface reactions, MOFs, with their large specific surface area, tunable structure, and a large number of active sites, are considered to be one of the most ideal sensing materials [118,119]. After the desired interaction between the analyte and the MOFs has been achieved, the MOFs’ physical and/or chemical properties can be altered to achieve detection purposes [120]. As an important branch of MOFs, luminescent metal–organic frameworks (LMOFs) have developed rapidly in recent years, featuring fast response, low detection limits, good selectivity, tunable structures, and a large number of active sites, etc. More particularly, most LMOFs used to detect analytes are reversible, which means that such products have good recyclability and low detection costs and have broad application prospects in the field of fluorescence sensing [121,122]. Typically, luminescence bursts and enhancements are how LMOFs achieve sensing detection of analytes. The mechanism of sensing and detection of various analytes by LMOFs can be divided into (1) MOF-analyte interactions, including hydrogen bonding and π–π interactions, affecting the energy transfer process; (2) effective filtering of excitation light by analytes adsorbed on MOFs (competitive adsorption); (3) collapse of the MOF framework structure induced by analytes and a break in the energy transfer process; (4) interaction of the guest molecule with the ligand, promoting the efficiency of energy conversion from the ligand to Ln^3+^ and increasing the luminescence intensity; (5) release of quenchers loaded in the luminescent MOFs and interaction with analytes inducing fluorescence enhancement [123,124].

**Figure 9:**
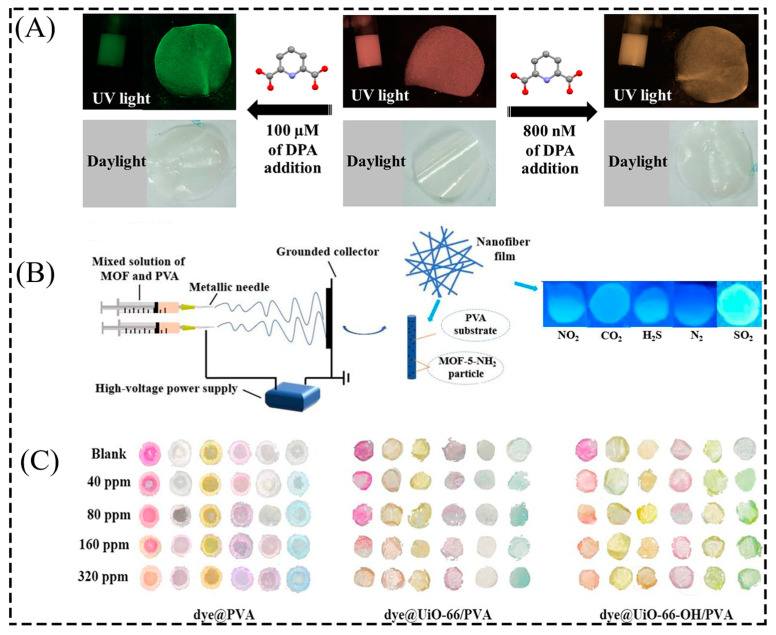
(**A**) Comparative photos of the luminescence of the solution phase and films without and with different levels of DPA added under UV and sunlight. (**B**) Comparative photos of the fluorescence of MOF-5-NH_2_/PVA nanofiber films in NO_2_, CO_2_, H_2_S, N_2_, and SO_2_. (**C**) Photos of the dye@UiO-66-OH/PVA composite films exposed to different concentrations of TMA gas. Reprinted with permission from Refs. [56,64,65]. Copyright 2020 Elsevier B.V. Copyright 2022 The Polymer Society. Copyright 2022 American Chemical Society.

However, most studies have focused on powdered MOF materials [125,126]. Combining MOFs with polymeric materials not only solves the problem of brittleness and instability of MOF particles but also improves their mechanical flexibility and ensures chemical stability [127]. Thin-film-based sensors have advantages over powdered MOF materials because they do not require separation and can be easily recycled. In addition, to eliminate the influence of the presence of aqueous solvents on the relative luminous intensity of each luminescent component to varying degrees, especially for white luminescent materials, the combination of PVA with MOFs shows higher water stability than pure MOFs at room temperature. Furthermore, the combination of PVA with MOFs enhances the accuracy and stability of the luminescent sensing [53], due to the energy transfer from the luminescent central ion Tb^3+^ to Eu^3+^ that occurs in Eu_0.002_Tb_0.018_-BDPO with increasing temperature, coupled with the good linearity of the emission intensity ratio (I_Tb_/I_Eu_) of the Tb^3+^ to Eu^3+^ transition with temperature. Wang et al. used Eu_0.002_Tb_0.018_-BDPO@PVA composite films as ratio luminescence thermometers with excellent temperature sensing performance in the temperature range of 303–423 K, enabling visible color changes with sensitivity up to 3.7% K^−1^. The emission spectra of Tb^3+^ and Eu^3+^ in [Tb_0.43_Eu_1.57_(1,4-phda)_3_(H_2_O)] (H_2_O)_2_ changed due to the presence of label Bacillus anthracis (pyridine dicarboxylic acid, DPA), indicating an energy transfer from DPA to the Tb^3+^ and Eu^3+^ centers and a linear relationship between the emission intensity ratio (I_Tb_/I_Eu_) and DPA concentration for DPA present in methanol and water. Therefore, Othong et al. used PVA/[Tb_0.43_Eu_1.57_(1,4-phda)_3_(H_2_O)] (H_2_O)_2_ composite films as a ratiometric luminescence sensor for the calibration of DPA. In the presence of DPA, the visible luminescence color of this composite film under UV light changes from red-orange in methanol to orange-yellow, and from red-orange in water to green (Figure 9A) [64].

In addition to combining Ln-MOFs with PVA to produce excellent membranes with sensing properties, Lou et al. [56] compared the fluorescence effect of MOF-5-NH_2_/PVA nanofiber membranes in the presence of NO_2_, CO_2_, H_2_S, N_2_, and SO_2_ gases. They found that the composite nanofiber membranes had a blue fluorescence effect only in the SO_2_ environment, which has a positive effect on the preparation of sensors with SO_2_ gas detection (Figure 9B). Ma et al. [65] prepared composite films of different colors (dye@UiO-66-OH/PVA, dye@UiO-66/PVA, and dye@PVA) by impregnating six dyes in MOF and PVA solutions. The sensing performance of the comparative composite films exposed to methylamine (MA) gas was investigated. With increasing MA gas concentration, dye@UiO-66-OH/PVA and dye@UiO-66/PVA show significant color differences due to the three-dimensional porous structure of UiO-66 particles, which enhances gas transfer and brings more reaction sites (Figure 9C). In addition, dye@UiO-66/PVA showed the lowest detection limit (80 ppm) and the shortest equilibration time (420 s). In addition, the composite membrane can also effectively identify gases such as NH_3_ and trimethylamine (TMA), which has great potential for food freshness testing.

### 3.3. Air Purification

Through risk factor studies, air pollution has been identified as the most significant contributor to disease and premature death in the world [128]. Ambient particulate matter (PM) is an environmental factor associated with increased respiratory morbidity and mortality. In susceptible populations, the main effect of PM on the cardiovascular system is to promote increased inflammation and thrombosis. The mechanism that induces its inflammatory and thrombogenic effects is the generation of oxidative stress by its compounds and metals [129,130]. In addition, the presence of toxic and hazardous gases in the air (e.g., volatile organic compounds (VOCs)) and acidic and alkaline gases (H_2_S, NH_3_) poses an alarming threat to public health and the environment, especially in some heavy industries and densely populated cities. Traditional adsorbents such as activated carbon and zeolite have been widely proven to be good candidates for removing toxic substances from the air, but most of these materials suffer from limited adsorption capacity and high regeneration energy consumption [131,132]. MOF particles are popular for the adsorption of airborne health hazards due to their unique and excellent properties such as large specific surface area, high porosity, easily designed pore structure, rich unsaturated metal site coordination, and the ability to tailor chemical functionality in a controlled manner [133,134,135]. For example, the metal ions of the MIL-100, MIL-101, and MOF-74 series allow for a variety of exchanges while maintaining the original topology [136,137,138]. Such a theoretically infinite number of metal ion–organic linker combinations makes them an unbeatable choice for targeted chemical interactions, allowing the adsorption of a wide range of chemicals [139]. For example, MIL-101 on acetone, benzene, toluene, ethylbenzene, xylene, methanol, dichloromethane, and other polar VOCs has a good adsorption effect [140]. Moreover, the adsorption capacity of MIL-101 for acetone, toluene, ethylbenzene, and xylene is also higher than that of commonly used adsorbents such as zeolite, activated carbon, and their derivatives [141]. MIL-101 has a large specific surface area and pore volume; moreover, when adsorbing benzene, the π–π interactions formed between benzene molecules and benzene rings in the ligand of MIL-101 are greater than the C–π and O–π interactions between benzene molecules and carbon materials or zeolites; therefore, the adsorption of benzene by MIL-101 at 288 K and 56 mbar is 16.7 mmol/g, which is almost zeolites (silica-1 and SBA-15) and 3–5 times more than activated carbon (Ajax and ACF) [142].

**Figure 10:**
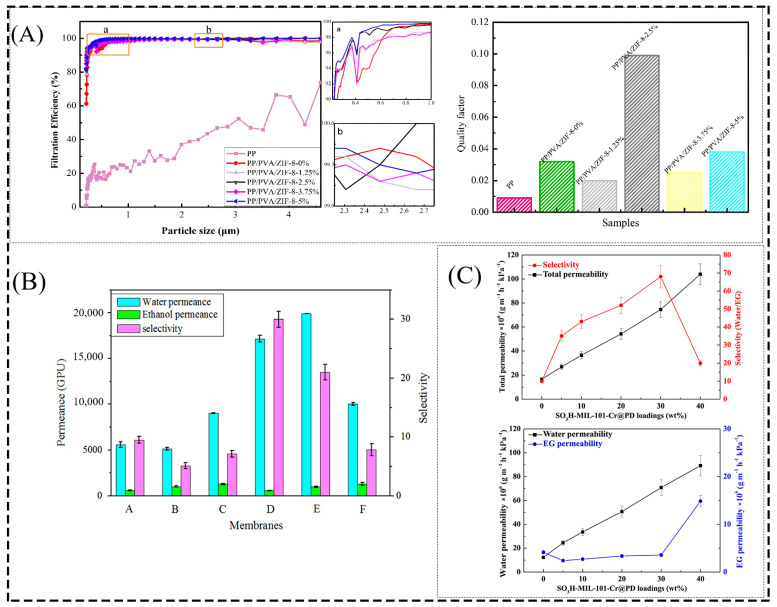
(**A**) Average filtration efficiency and quality factor of PP/PVA/ZIF-8 composite membranes for PM_2.5_. (**B**) Effect of 92.5 w% ethanol dehydration at 40 °C for different types of MOF composite membranes. (**C**) Effect of SO_3_H−MIL-101−Cr@PD−PVA hybrid membranes for ethylene glycol dehydration at different loadings. Reprinted with permission from Refs. [51,66,67]. Copyright 2020 MDPI AG. Copyright 2016 Elsevier B.V. Copyright 2017 Elsevier B.V.

MOFs can be put to better use in practice to solve the problems of difficult powder recovery and avoiding secondary contamination and to improve their chemical stability and make them more durable under extreme conditions. Combining MOFs with polymeric materials to develop flexible film materials can be used for more efficient and portable air purification applications, with PVA, a non-toxic, non-hazardous, biodegradable, and good film-forming substance, being a reliable choice. Typically, MOF-based composite membranes interact with contaminants by (1) binding to the open metal sites of MOFs; (2) interacting with functional groups on MOFs or polymers; and (3) electrostatic interactions with MOF nanocrystals [143]. In particular, MOF/polymer fibrous membranes prepared by electrostatic spinning can exhibit higher surface roughness, larger specific surface area, and porosity, increasing the probability of fibrous membrane collisions with contaminants, which will facilitate the capture of PM and toxic gases [144,145]. Compared with the SiO_2_ nanofiber membrane, the ZIF-8@SiO_2_ composite nanofiber membrane increased the electrostatic interaction with PM due to the increased surface area and the unbalanced metal ions and defects of MOF, and the removal efficiency of PM was as high as 99.96%, and the adsorption of formaldehyde also increased with the increase in ZIF-8 content on the composite nanofiber, up to 48.87 mg/g, while the adsorption of formaldehyde on the SiO_2_ nanofiber membrane was 1.49 mg/g [146]. The PP/PVA/ZIF-8 composite membrane prepared by Li et al. [66] had a positive effect on the adsorption of nanoparticles on the fiber surface due to the increased specific surface area of the composite membrane when loaded with 2.5% ZIF-8, and the average filtration efficiency of the PP/PVA/ZIF-8 composite membrane for PM_2.5_ was as high as 96.5% with a quality factor (Q_f_) of 0.099 Pa^−1^ (Figure 10A). Kim et al. [147] used a combination of electrostatic spinning and electrospray processes to prepare MOF/PVA nanofiber (NF) hybrid membranes on polyethylene terephthalate nonwovens (NWs). This MOF+NF/NW had a maximum filtration efficiency of 97% for air and demonstrated excellent gas capture efficiencies of 60% and 35% lower than the initial NH_3_ and H_2_S concentrations due to the reduction in pore size of the hybrid membrane and the interaction of contaminants with the functional groups on the MOF surface. The HKUST-1-PAA-PVA and HKUST-1-citric acid-PVA membranes prepared by Truong et al. showed that NH_3_ was removed due to adsorption by the lattice of HKUST-1 and interaction with the carboxyl groups on the nanofiber surface via hydrogen bonding and/or ionic interactions. The composite membranes achieved 85% and 87% removal of NH_3_, respectively [59].

### 3.4. Separation

With the depletion of fossil fuels, the development of clean, renewable biofuels has entered the limelight because of their ability to alleviate the global energy crisis and environmental degradation, especially renewable clean biofuels such as bioethanol [148,149]. The corresponding separation technology is pervaporation (PV), which is highly selective, efficient, and energy-saving and is widely used for the dehydration of organic solvents, the separation of organic–organic mixtures, and the removal of dilute organic compounds from aqueous systems [150,151]. The choice of hydrophilic membrane is key when dealing with the dewatering and separation of any organic substance. Currently, hydrophilic polymers such as polyimide [152], polysulfone (PSF) [153], CA [154], PAN [155], polyvinylamine (PVAm) [156], and PVA [157] are being investigated as film-forming materials. PVA is widely used in organic solvent dehydration due to its high chemical stability, hydrophilicity, low cost, excellent film-forming properties, and good mechanical properties, and it is also the only polymer that cures on an industrial level. To improve the separability and selectivity of polymeric membranes, the introduction of suitable inorganic materials into the polymer matrix is a very successful trend [158]. Among porous inorganic materials, the most typical examples are zeolites and carbon-based materials. Because of their amorphous structure, activated carbons are popular among porous inorganic materials due to their higher porosity and specific surface area compared to zeolites, despite their inhomogeneous structure. The emergence of new porous materials has been accompanied by excellent properties, such as MOFs, which combine the synergistic properties of organic–inorganic materials and have a molecular sieve effect, a large specific surface area, and a uniform and well-defined pore size, as well as a tunable structure. They show great promise in separation applications. Over the past decade, MOFs have been extensively investigated for separation applications, including the separation and capture of many gases and vapors and phase separation including liquid mixtures. MOFs also present new opportunities for enantioselective separations and are membranes that easily enhance separation results, showing potential to become a valuable building block in the manufacture of high-performance PV membranes [159,160]. The mechanism used to achieve the separation of MOF-based MMMs by PV technology is mainly the molecular sieving effect and/or the preferential adsorption of certain compound molecules by MOFs. In the former separation mechanism, molecules in the liquid mixture pass through or are blocked in the MOFs depending on the size of the pores of the MOFs [161]. In the latter mechanism, the preferential adsorption of target molecules by the MOF is utilized as the pores of the MOF that are sufficiently large or structurally flexible to allow the passage of molecules from all liquid mixtures. The interactions of the target molecules with the surface of the MOFs influence the properties of the MMMs, and these interactions (Eint = μ·E_x,y,z_) depend on the permanent dipole moment of the polar molecule (μ) and on the electric field strength determined by the local space charge density of the MOF (E_x,y,z_) [160].

The performance of MOF-based MMMs in the separation of liquid mixtures is usually evaluated using selectivity and water permeability. Wu et al. [67] used modified MIL-53(Al)-NH_2_ nanocrystals, added to PVA membranes for the total evaporation of 92.5 wt% ethanol. Due to interparticle interactions (e.g., hydrogen bonding, π–π interactions), the best MIL-53-NHCOH/PVA MMMs showed a 206% increase in water permeability and a 200% increase in selectivity, and the best MIL-53-NHCOC_4_H_9_/PVA MMMs showed a 340% increase in water permeability and a 170% increase in selectivity compared to the pristine PVA membrane (Figure 10B). Similarly, the group prepared MOF particles containing different organic ligands and incorporated them into PVA membranes for the total evaporation of 90 wt% ethanol. Compared to pristine PVA membranes, UiO-66-(OH)_2_/PVA MMMs loaded at 1.0 wt% showed 24% and 10% improvement in water permeability and selectivity, respectively, and a 28% reduction in swelling, due to the enhanced hydrophilicity of MMMs and hydrogen bonding of PVA with Zr-MOF ligands, which favored their selectivity for water adsorption and diffusion and showed a high potential for dehydration in ethanol solutions [162]. In addition to the application of MOF/PVA MMMs to ethanol dehydration by the PV technique, due to the PD containing a large number of hydrophilic groups encapsulated on the surface of SO_3_H-MIL-101-Cr with a macroporous structure and good compatibility between the polymer and filler, the well-dispersed nano-charges produced a diffusion limitation for the bulkiest molecules. Zhang et al. investigated the prepared SO_3_H-MIL-101-Cr@PD-PVA MMMs with good water permeability, separating glycols containing 10 wt% water at 343 K with water permeability and selectivity up to 7.0 × 10^−5^ g m^−1^ h^−1^ kPa^−1^, 68.1 (separation factor of 2864), which is 483% and 567% higher than the water permeability and selectivity of the original PVA membrane, respectively (Figure 10C) [51]. Amirilargani et al. [68] prepared a PVA/ZIF-8 hybrid membrane for the separation of isopropanol. The hybrid membrane with an optimum loading of 5 wt% ZIF-8 improved the permeability coefficient by a factor of five compared to the original PVA membrane.

### 3.5. Antibacterial

Diseases caused by the transmission of pathogenic microorganisms have become one of the greatest threats to the global public health system, such as SARS and COVID-19 [163,164]. To fight against these pathogenic microorganisms, the resistance of pathogenic bacteria increases after overuse of antibiotics, which makes the treatment of antibiotics extremely difficult. Therefore, the development of new antibacterial materials has been in full swings, such as metal/metal oxide nanoparticles [165], nano enzymes [166], and semiconductor photocatalytic materials [167]. As a kind of MOF material with good features such as high specific surface area, high enzyme-like activity, strong interaction with bacterial membranes, high drug loading capacity, and continuously controlled release of metal ions, it has gained a lot of attention in various antimicrobial applications [168]. Moreover, MOFs have the same or similar active centers as in the very metal/metal oxide nanoparticles, which are moderately strong, robust, and flexible. Given that the antimicrobial effect of the presence of metal ions (e.g., silver, zinc, and copper) in MOFs is associated with physical damage to bacterial cells, i.e., the release of metal ions that can cross cell membranes and lead to cell destruction, MOFs hold promise as a support or alternative to antibiotics [169,170]. The organic ligands in the MOF structure can act both as antibiotics and as photosensitizers that produce reactive oxygen species (ROS) to kill bacteria upon light irradiation [171]. In addition, the advantage of MOFs as new high-performance antimicrobial materials is that the internal surface area of MOFs is large enough to be used as a storage site for antimicrobial agents [172]. MOFs provide a diverse platform for antimicrobial materials through the formation of nanocomposites between MOFs and polymers that can perform functions not possible with individual components, including controlled polymerization and new stable composite films. For example, Seyedpour et al. [173] reported a polyamide-positive permeable thin-film composite (TFC) by in situ depositing Ag-MOF. The confocal microscopy showed that Ag-MOF gave the membrane a strong antibacterial performance with a nearly 100% reduction in viable bacteria. MMMs prepared by Liu et al. [174] compounding Zr-MOF and poly(ε-caprolactone) (PCL), irradiated by LED, produced ROS that acted as efficient photosensitizers to inhibit *E. coli*.

**Figure 11:**
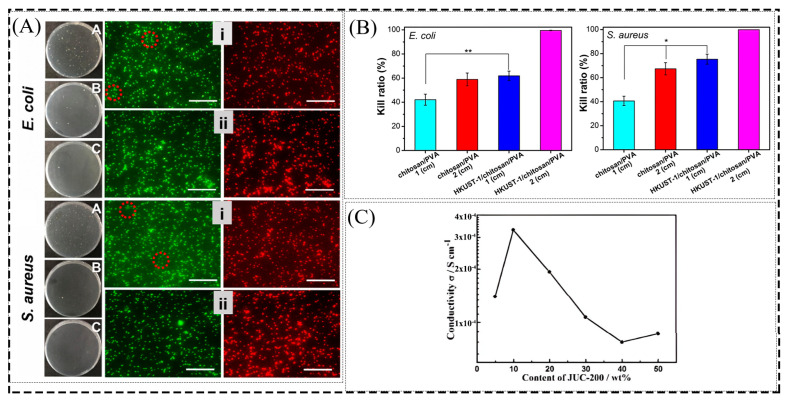
(**A**) Colony plots for live/dead bacterial viability assay of *E. coli* and *S. aureus*. A: UiO-66-NH_2_@PVA at a concentration of 50 μg/mL, B and i: LV@UiO-66-NH_2_@PVA at a concentration of 50 μg/mL, C and ii: LV@UiO-66-NH_2_@PVA at a concentration of 100 μg/mL (**B**) Killing rate of *E. coli* and *S. aureus* by colony counting. * *p* < 0.05, ** *p* < 0.01. (**C**) The conductivity of composite membranes with different JUC-200 content at 20 °C. Reprinted with permission from Refs. [57,58,71]. Copyright 2022 Elsevier Inc. Copyright 2020 Elsevier B.V. Copyright 2017 The Royal Society of Chemistry.

In addition to the low toxicity, biodegradability, and good dispersion of MOF particles combined with strong interfacial interactions with non-toxic and non-hazardous PVA polymeric materials, MOF-based MMMs with high antimicrobial efficiency have great potential for applications in the healthcare sector. The three-dimensional network structure of the fiber membrane facilitates the adsorption of bacteria. Singbumrung et al. [69] studied the antibacterial activity of Cu-BTC/PVA electrospun fibers against *Escherichia coli* (*E. coli*) and Staphylococcus aureus (*S. aureus*) and concluded that Cu-BTC/PVA electrospun fibers have strong antibacterial properties due to the interaction between the released Cu^2+^ and the bacterial membrane. The antibacterial activity increased with increasing concentration of Cu-BTC and completely inhibited the growth of Staphylococcus aureus. Zhu et al. [58] studied the antibacterial activity of UiO-66-NH_2_@PVA nanofiber membranes compared with LV@UiO-66-NH_2_@PVA nanofiber membranes loaded with the antibacterial drug levofloxacin (LV) against *E. coli* and *S. aureus*. Due to the long-term effective release of LV stored in the pores of the nanofiber membrane and UiO-66-NH_2_, the antibacterial performance of the nanofiber membrane was enhanced, especially the bactericidal efficiency of LV@UiO-66-NH_2_@PVA at 100μg/mL, which was more significant than 99.9% for both *E. coli* and *S. aureus* (Figure 11A). In addition, the composite membrane without adding antibacterial agents can also show a good antibacterial effect. Wang et al. [57] prepared HKUST-1/chitosan/PVA nanofiber membranes by electrostatic spinning, which, in addition to exhibiting good physical properties, also had good biocompatibility, and the composite fiber membrane also had over 99% antibacterial properties due to the release of Cu^2+^ (Figure 11B). Thanks to the adequate enrichment and antimicrobial activity of volatile amines, MIL-101 is a promising material. Lin et al. [70] introduced cheap and readily available grape skin anthocyanins (GSAs) and MIL-101 into a starch/PVA (PS) film. They prepared a composite film that exhibited good antibacterial properties and helped maintain the pork’s freshness.

### 3.6. Other Applications

The world’s demand for electric energy devices has accelerated the development of new energy storage systems with high energy density and long life [175]. For the fuel cell as a new type of energy storage device that has received significant attention, the development of high proton conductivity membrane materials can solve the problems of high cost and mild working conditions of commercial proton exchange membrane materials [176,177]. The JUC-200@PVA composite membrane with a loading capacity of 10 wt% prepared by Cai et al. [71] had a good proton conductivity of 1.25 × 10^−3^ S·cm^−1^ at 50 °C (Figure 11C). Yang et al. [178] added the prepared Ln-MOF to a PVA matrix to obtain a composite membrane with better proton conductivity than Ln-MOF alone under the same conditions. In addition, it is vital to prepare materials with high UV-barrier properties, as polymers’ performance and service life can be affected by high-energy UV radiation [179]. Our group compared the UV-blocking performance of HKUST-1/PVA composite film, ZnO/PVA composite film, and UV531/PVA composite film [72]. It showed that the 5 wt% loading of HKUST-1/PVA composite film exhibited up to 98.8% UV-blocking performance with better water resistance and mechanical properties.

## 4. Conclusions and Outlook

The combination of MOF materials with high specific surface area, regular pore size, and tunable structure with PVA, a biodegradable material with low toxicity, cheap and easy availability, good mechanical properties, and biocompatibility, is an important material for the preparation of composite membranes with multiple application potentials. This paper summarizes the preparation of MOF/PVA composite films from PVA and MOFs using solution casting, electrostatic spinning, and other different methods. The simple and effective embedding of MOF particles in PVA membrane materials by these methods has led to widespread interest in MOF/PVA composite membrane materials for a wide range of applications such as water purification, sensing, air purification, antimicrobials, UV blocking, and so on. However, to further achieve the functionalization of MOF/PVA composite membranes, the prepared composite membranes still face some challenges in improving the dispersion of MOF particles in PVA, active sites, and specific surface area. In the future, the experimental methods can be further optimized, e.g., pre-dispersing MOF particles ultrasonically in solvents, preparing good quality MOF particles, designing organic ligands for specific functions, optimizing electrostatic spinning parameters, exploring the viscosity of the PVA solution, and incorporating dispersing agents or cross-linking agents, etc., to improve the uniform and stable loading of MOF particles on the composite membranes and to increase the specific surface area of the composite membranes with the active sites, which is crucial for evaluating the performances of the MOF/PVA composite membranes. In addition, this paper concludes that MOF/PVA composite membranes are found to have important application potential in utilizing their adsorptive separation properties; therefore, more optimization of the process of MOF/PVA composite membranes as an adsorptive separation application can be investigated in the future. Finally, to give more excellent properties to MOF/PVA composite membranes, MOFs or PVA can be pre-combined with different functional materials, and then the structure–property relationship of MOF/PVA composite membranes combined with functional materials can be investigated to explore more different applications, as different applications may require composites with different properties.

## Figures and Tables

**Table 1:** MOF/PVA composite films with different MOF particles and their applications.

MOF	Preparation Methods	Applications	Ref.
UiO-66-NH_2_	Solution casting	Gas separation	[44]
HKUST-1	Solution casting	UV blocking	[45]
UiO-66-X	Solution casting	Separation	[46]
ZIF-8	Solution casting	Ionic dynamics	[47]
ZIF-8	Solution casting	---	[48]
ZIF-90	Solution casting	Separation	[49]
ZIF-8	Solution casting	Water treatment	[50]
SO_3_H-MIL-101-Cr	Solution casting	Separation	[51]
Cu-BTTri	Solution casting	Healthcare	[52]
MOF-76(Eu_x_Tb_1−x_)	Solution casting	Sensing	[53]
ZIF-90	Solution casting	Separation	[54]
ZIF-8	Solution casting	Electrochemistry	[55]
MOF-5-NH_2_	Electrospinning	Sensing	[56]
HKUST-1	Electrospinning	Antibacterial	[57]
UiO-66-NH_2_	Electrospinning	Antibacterial	[58]
HKUST-1	Electrospinning	Air purification	[59]
UiO-66	Spraying method	Water treatment	[60]
Zn-MOF	Spin coating	Electrochemistry	[61]
UiO-66-NH_2_	Vacuum filtration	Water treatment	[62]
ZIF-8	Electrospinning	Water treatment	[63]
Ln-MOF	Solution casting	Sensing	[64]
UiO-66-X	Solution casting	Sensing	[65]
ZIF-8	Electrospinning	Air purification	[66]
MIL-53(Al)-NH_2_	Solution casting	Separation	[67]
ZIF-8	Solution casting	Separation	[68]
Cu-BTC	Electrospinning	Antibacterial	[69]
MIL-101	Solution casting	Antibacterial	[70]
JUC-200	Solution casting	Electrochemistry	[71]
HKUST-1	Solution casting	UV blocking	[72]

**Table 2:** Comparison of preparation methods of MOF/PVA composite membranes.

Synthesis Method		Advantages	Disadvantages
Solution Casting	Blending	Convenient and fast	Agglomerates easily
		Low operation cost	
	In situ growth	Even distribution Good interface compatibility	High MOF loads are difficult to achieve
Electrospinning	Blending	High specific surface area, high flux, high porosity	Low efficiency in mass production
	Layer-by-layer assembly	High specific surface area and high active site Multi-layer assembly, better performance	The interface between layers may be defective Time consuming
Spraying	Blending	Even distribution Convenient and fast Large-scale production	High dispersion requirements for suspension Uniformity and thickness are difficult to control
Spin coating	In situ growth	The airflow during the rotation is conducive to uniform drying	Low concentration solution is difficult to coat Not suitable for mass production
Vacuum filtration	Blending	Convenient operation Easy to control film thickness	Not suitable for mass production The film is easily damaged during the separation process

## Data Availability

Not applicable.
